# Supplementary figures and images for: The Association Between Alpha-1 Adrenergic Receptor Antagonists and In-Hospital Mortality From COVID-19
Source: Front Med (Lausanne). 2021 Mar 31;8:637647. doi: 10.3389/fmed.2021.637647 (PMC8048524; doi:10.3389/fmed.2021.637647)

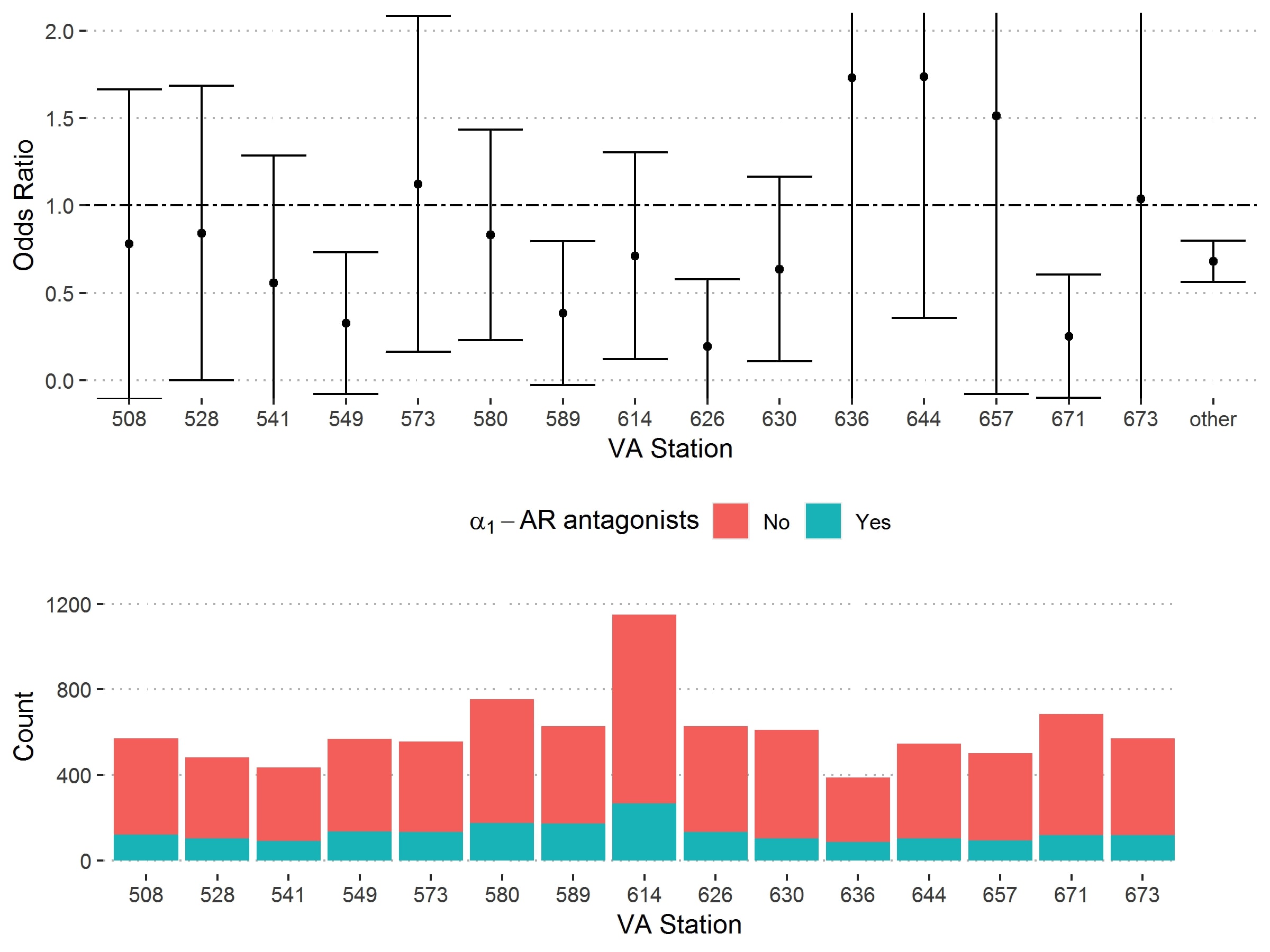

Supplement: Supplementary Figure 1 — Vital Signs at Time of Admission. The diagrams show vital signs for patients diagnosed with COVID-19 (red line) and an expanded cohort of patients with suspected COVID-19 (blue line). Smoothed lines are from a LOESS model with 95% confidence intervals shown (gray ribbons). [file Image_1.JPEG]

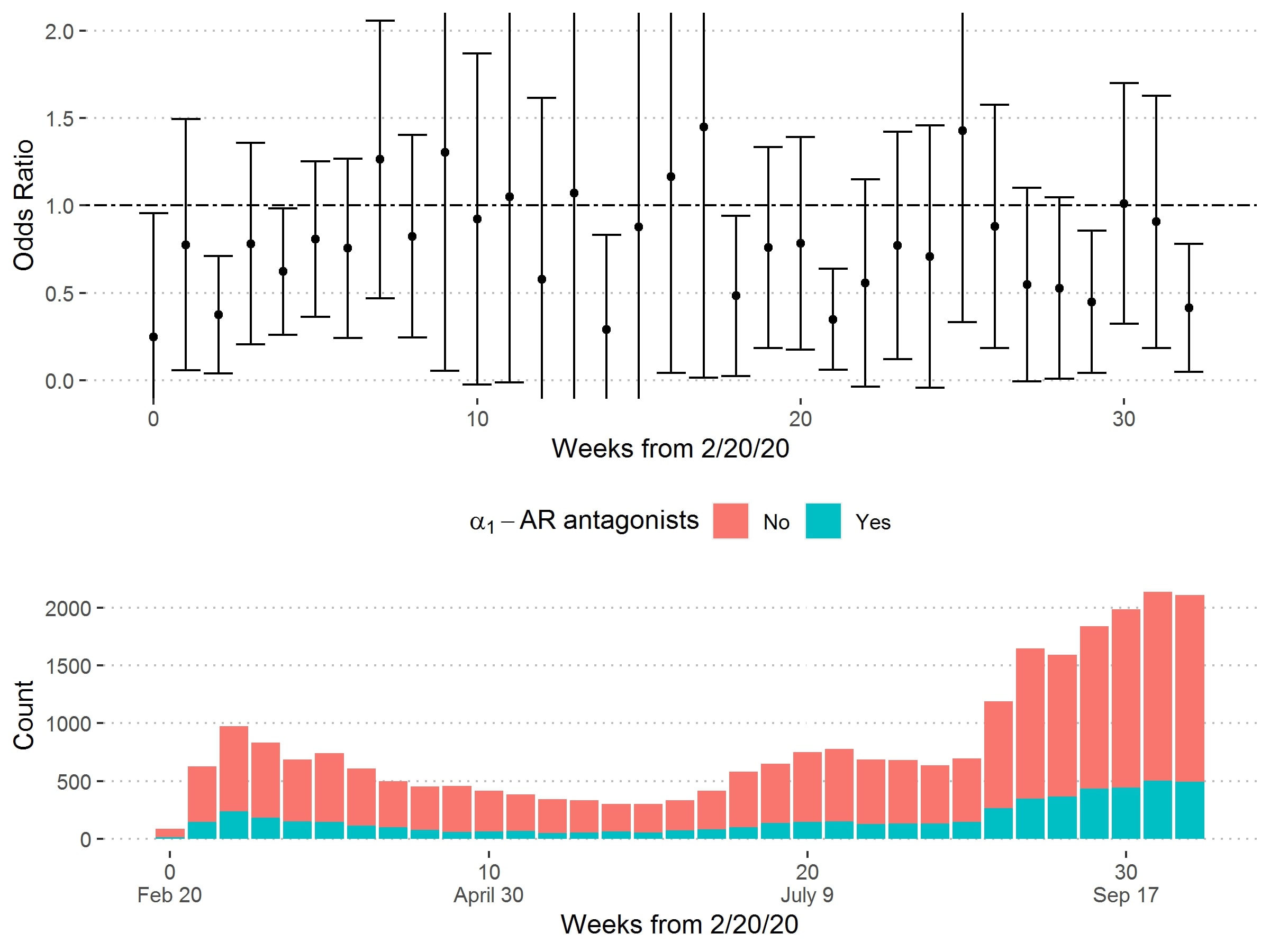

Supplement: Supplementary Figure 2 — In-hospital and 28-Day Mortality by Use of Tamsulosin at Time of Hospital Admission with COVID-19. Data are shown for hospitalized patients diagnosed with confirmed COVID-19 (top panel) and with confirmed plus suspected COVID-19 (bottom panel). Forest plots showing odds ratios (OR) of in-hospital mortality based on prior use of any alpha-1 adrenergic receptor antagonists (dark green) or tamsulosin (light blue) in each panel. Relative risk reduction (RRR), odds ratios (ORs) for death, 95% confidence intervals (CI), and p-values (for unadjusted, adjusted, and matched models), and sample size (n) for each analysis are shown on the right. [file Image_2.JPEG]

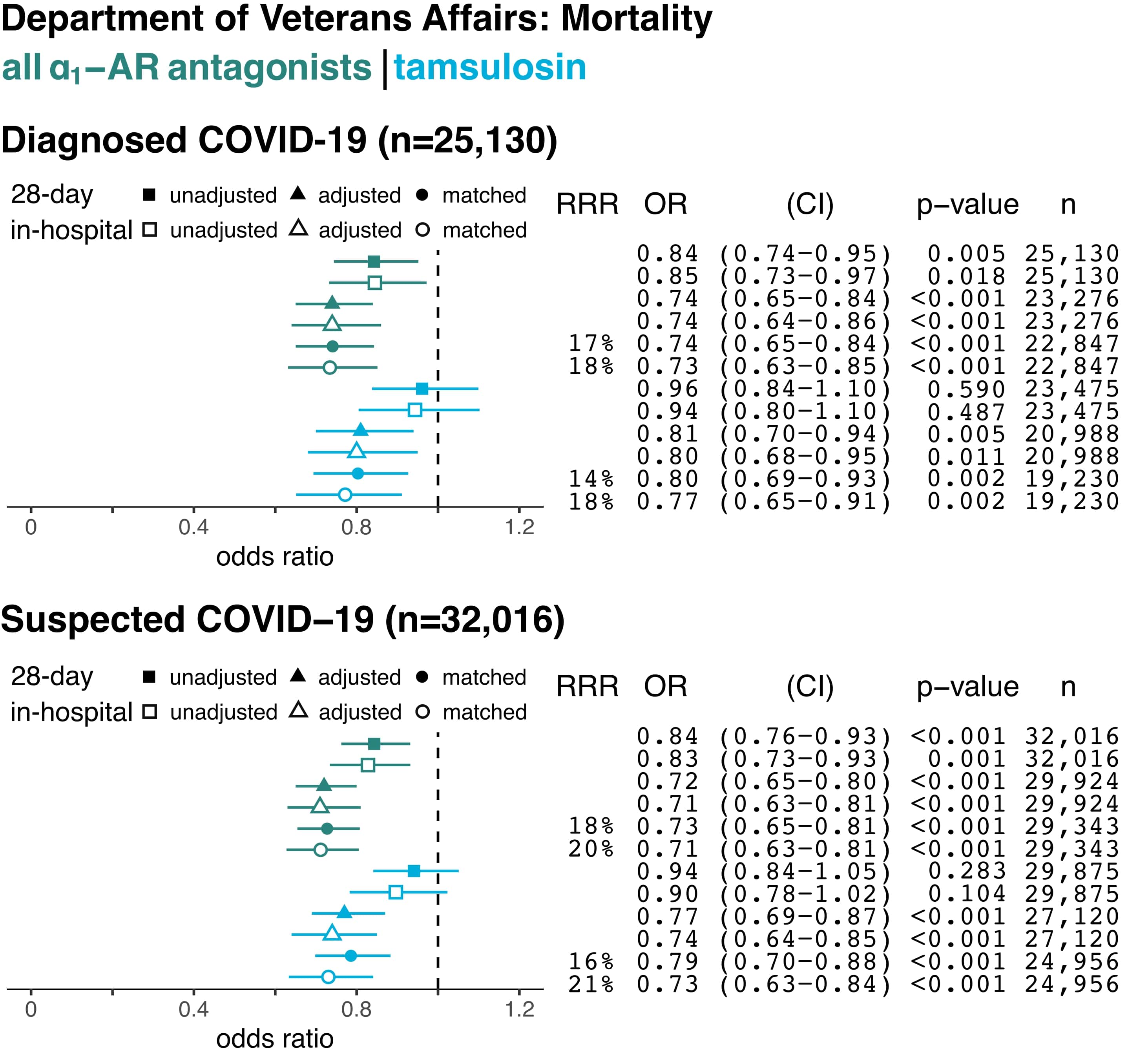

Supplement: Supplementary Figure 3 — Adjusted Odds of In-hospital Mortality and Use of α1-AR Antagonists by Week. Top panel shows adjusted odds ratios of in-hospital mortality and use of α1-AR antagonists by week of admission. Top panel truncated between 0 and 2 to aid visualization. Bottom panel shows number of new admissions by week and use of α1-AR antagonists (bottom). [file Image_3.JPEG]

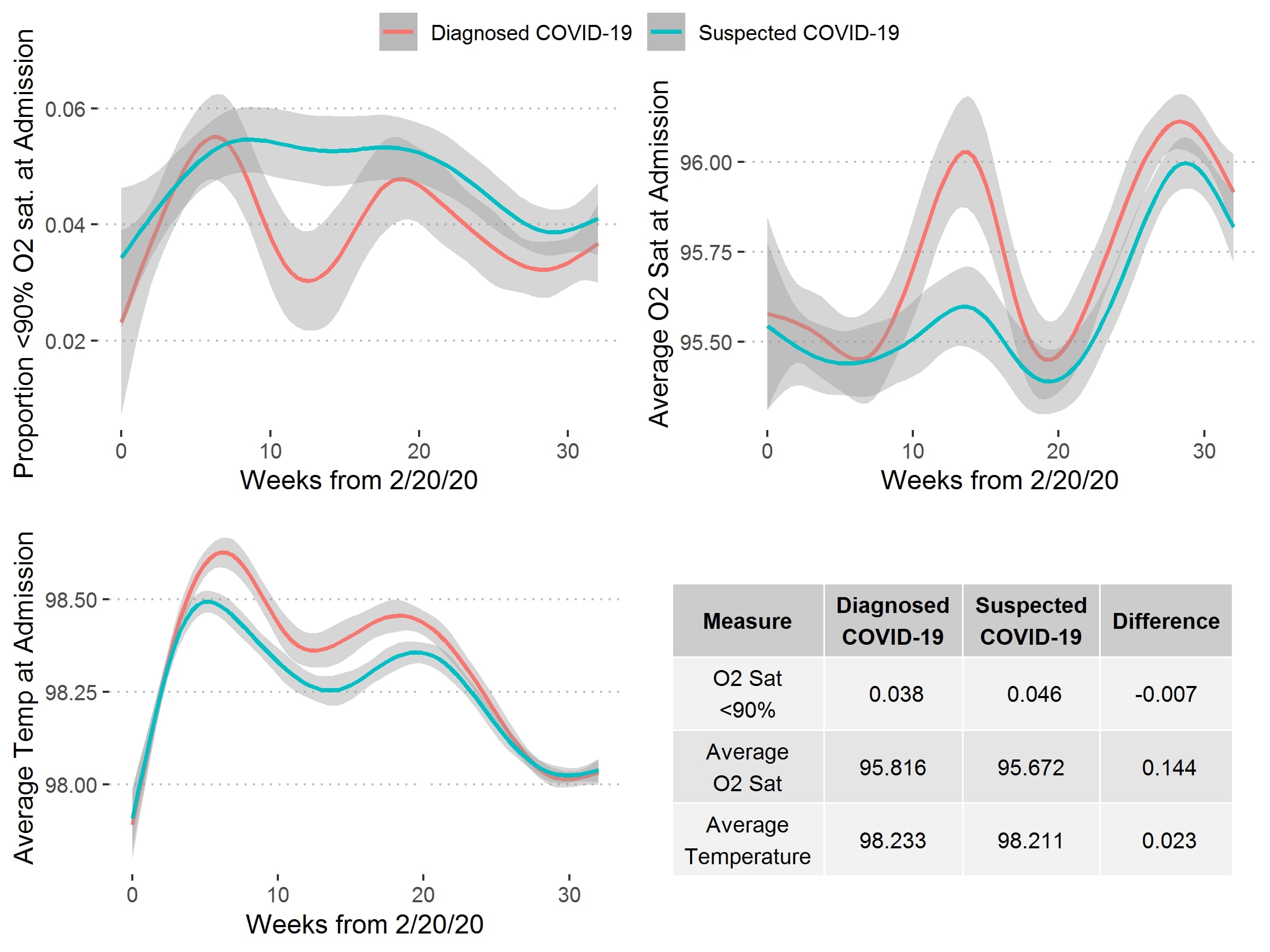

Supplement: Supplementary Figure 4 — Adjusted Odds of In-hospital Mortality and Use of α1-AR Antagonists by VA Station. Top panel shows adjusted odds ratios of in-hospital mortality in patients taking α1-AR antagonists by VA station. Top panel truncated between 0 and 2 to aid visualization. Bottom panel shows number of new admissions and use of α1-AR antagonists by VA station (bottom). For other VA stations, the number of admissions of patients not using α1-AR antagonists was 7,645 and number of admissions of patients using α1-AR antagonists was 1,845. VA stations shown: 508 = Atlanta, 549 = Dallas, 573 = Gainesville, 580 = Houston, 589 = Kansas City, 614 = Memphis, 630 = New York Harbor, 644 = Phoenix, 671 = San Antonio, 673 = Tampa. [file Image_4.JPEG]
